# Abdominal Ultrasonography Used for Abdominal Pain in the Rural Outpatient Setting of South Texas: Impact on Patient Outcomes

**DOI:** 10.7759/cureus.64462

**Published:** 2024-07-13

**Authors:** Elizabeth Mills-Reyes, Kathryn N Devlin, Pablo Olmedo

**Affiliations:** 1 Family and Community Medicine, Oceania University of Medicine, Mission, USA; 2 Research, Drexel University, Philadelphia, USA; 3 Family Medicine, Sagrado Corazon Family Clinic, Mission, USA

**Keywords:** abdominal pain and uninsured, abdominal pain and imaging, abdominal pain and hispanic/latino, abdominal pain and outcomes, abdominal pain and abdominal ultrasonography

## Abstract

Introduction

Abdominal ultrasonography is a key diagnostic tool used in complaints of abdominal pain. The rationale for this study is to examine abdominal ultrasonography’s impact on the conclusion of care of abdominal pain in a predominantly Hispanic/Latino patient population.

Materials and methods

A chart review of 350 patients with a new diagnosis of abdominal pain from a rural family practice clinic in Texas was performed. These patients' charts were reviewed for a new diagnosis of abdominal pain, medications prescribed for abdominal pain, whether abdominal ultrasonography was completed, and the number of visits regarding their complaint. The last visit for their abdominal pain was denoted as the conclusion of care of abdominal pain within the clinic. The primary analyses were logistic regressions with conclusion of pain care or number of visits as the outcome and abdominal ultrasound completion as the primary predictor.

Results

The sample size was 216 of the 350. Patients were excluded due to age under 18 and if the patient's pain was not coded as epigastric, generalized, or right upper quadrant pain. The patient age range was 18-88 years, and they were all of Hispanic/Latino origin. Abdominal ultrasound was completed on 59 of the patients, and 65 patients experienced conclusion of primary care for abdominal pain. Regarding the number of visits for abdominal pain, 69% had one visit, 25% had two visits, and 6% had three or more visits. Patients who had abdominal ultrasounds were more likely to have multiple visits (typically just two visits) but had markedly higher conclusions of care for abdominal pain. These relationships remained when adjusting for demographic and medical covariates such as age, abdominal pain (all types), and medical treatments used.

Conclusion

In the outpatient rural care of Hispanic/Latino patients residing in the Rio Grande Valley, patients who had a new complaint of abdominal pain were more likely to have conclusion of primary care for abdominal pain, with only a slight increase in primary care healthcare consumption, if abdominal ultrasonography was completed for abdominal pain.

## Introduction

In emergency rooms, 5-10% of the visits are due to abdominal pain [1]. Due to the numerous causes of abdominal pain and the risks of adverse outcomes if misdiagnosed, primary care providers have an invaluable task of rooting out causes and ensuring no life-threatening causes arise [1]. A detailed history and physical examination are paramount when evaluating patients with abdominal pain no matter the setting. In primary care, when targeted imaging is done, it is often ultrasound initially [2]. Because of the complexities of abdominal pain, imaging is not only often indicated and frequently used in the emergency room but also utilized in primary care to exclude or confirm diagnoses [3]. Although computed tomography scan is often a first-line diagnostic procedure in the hospital, this is not always available and is often less utilized by primary care [2,4].

One study evaluated bedside surgeon abdominal ultrasonography in the emergency department setting, which demonstrated better patient satisfaction and less consumption of healthcare in the short term [5]. Patients’ understanding of what diagnostics can demonstrate is limited, and patients may feel relieved when testing has been completed even if their understanding of the test is limited. Patients may demand imaging to resolve some of their concerns or anxiety about their illness or pain [6]. On the other hand, there is no conclusive evidence that diagnostic imaging intrinsically assuages a patient's fears about their medical concern [7]. Despite these studies noting the impact of imaging on the patient's perception or satisfaction, these researchers did not identify any other studies that show if visits for abdominal pain are affected by abdominal ultrasonography in primary care.

It is anticipated that by 2060, more than 25% of the United States will be Hispanic/Latino [8]. Presently 90% of the population of the Rio Grande Valley of Texas is Hispanic [9]. Despite the rising national population of Hispanic/Latino persons, medical research demonstrates a disparity in the amount of Hispanic/Latino-focused research [8].

The current study was designed to investigate abdominal pain in the rural South Texas primary care setting of a predominantly Hispanic/Latino practice of the Rio Grande Valley and how abdominal ultrasound impacts 1) whether primary care for abdominal pain is concluded and 2) the number of visits that the patient undergoes for abdominal pain. This researcher’s hypothesis is that patients who undergo abdominal ultrasonography are more likely to have fewer subsequent visits for abdominal pain than those who do not have abdominal ultrasonography within this population.

## Materials and methods

This is a retrospective study of 350 patients in a family practice clinic in Mission, Texas, from January 2019 through May 2023 with a new diagnosis of abdominal pain. Inclusion criteria were patients with a new diagnosis of abdominal pain, limited to diagnosis codes for generalized abdominal pain (International Classification of Disease [ICD-10] code R10.9), right upper quadrant (RUQ) pain (ICD-10 code R10.11), and epigastric pain (ICD-10 code R10.13). Because diagnosis code R10.13 can also be used for dyspepsia not otherwise specified, the progress notes were required to include specific notation of pain in the patient's history of present illness. Patients were excluded if they were younger than 18 years or if they had other types of abdominal pain or pelvic pain. Based on the above criteria, only 216 patients met the criteria for this study.

This study was approved by the Oceania University of Medicine Institutional Review Board (OUMHREC24_016).

The following information was extracted from patient charts from the time of the initial visit for abdominal pain through six months later: diagnosis code of the presenting problem, age, gender, ethnicity, insurance status, initial medications prescribed, whether an abdominal ultrasound was ordered, whether abdominal ultrasound was performed, abdominal ultrasound results, number of primary care visits for abdominal pain, and whether primary care for abdominal pain care was concluded (i.e., cessation of office visits for abdominal pain). If patients required referral to a specialist or if they underwent surgery for abdominal pain, this was noted.

Statistical analysis

Statistical analysis was performed using IBM SPSS Version 29 (IBM Corp., Armonk, NY) [10]. Descriptive statistics for all variables were computed for the whole sample. In inferential analyses, the primary independent variable was abdominal ultrasonography completion. The primary outcome was the cessation of office visits for abdominal pain. The number of visits for abdominal pain was a secondary outcome. Covariates of interest included age, gender, insurance status (insured versus uninsured), diagnostic code of presenting problem (unspecified abdominal pain, RUQ pain, epigastric pain), and initial medical management (none, analgesic, gastrointestinal [GI], both analgesic and GI, other). These covariates were examined in relation to each other, abdominal ultrasound completion, and cessation of visits for abdominal pain using chi-squared tests, Fisher’s exact tests, or analysis of variance (ANOVA), as appropriate. The primary analysis was a hierarchical logistic regression with pain resolution as the outcome. Abdominal ultrasonography completion was added as a predictor in step 1, followed by covariates in step 2. A secondary logistic regression analysis was conducted with number of visits (2+ vs. 1) as the outcome. Alpha level was set at 0.05 for all analyses.

## Results

Sample characteristics 

Sample characteristics are presented in Table 1; 216 patients met inclusion criteria, including 90 (42%) with unspecified abdominal pain, 69 (32%) with RUQ pain, and 57 (26%) with epigastric pain. Age ranged from 18 to 88 years, with an average age of 43 years. Most patients were women (66%; 143/216), an overwhelming majority were Hispanic (all but one), and 43% (92/216) were uninsured. In addition, 81% (197/216) of the patients were prescribed some form of medical management at the initial visit, usually GI medication, followed by analgesic medication or a combination thereof. Abdominal ultrasound was ordered in 70 (32%) patients; 59 (27%) completed the ultrasound, and 11 (5%) did not. Among those who completed it, 64% (38/59) had a positive result, usually gastrointestinal. Most patients had only one visit for abdominal pain. A large minority or 25% (53/216) of the patients had two visits, and few had three or more visits. Primary care visits for abdominal pain care were concluded in 30% (65/216) of patients, usually either through initial medical management or referral for surgical or specialty care.

**Table 1:** Sample demographic and clinical characteristics and outcomes US, ultrasound; RUQ, right upper quadrant; GI, gastrointestinal; UTI, urinary tract infection; BPH, benign prostatic hyperplasia

Demographics and Presenting Problem		N	%
Presenting abdominal pain problem	Unspecified (R10.9)	90	42%
	RUQ (R10.11)	69	32%
	Epigastric (R10.13)	57	26%
Age		M=43	SD=16
Gender	Female	143	66%
	Male	73	34%
Ethnicity	Caucasian	1	0.5%
	Hispanic	215	99.5%
Insurance	Uninsured	92	43%
	Insured	124	57%
Outcomes of initial visit			
Abdominal US status	Not ordered	146	68%
	Ordered but not completed	11	5%
	Completed	59	27%
Abdominal US result if completed	Negative	21	36%
	Positive	38	64%
Abdominal US results category if positive	GI	30	79%
	Renal	6	16%
	Other	2	5%
Medical management	None	40	19%
	Analgesic	35	16%
	GI	104	48%
	Analgesic and GI	22	10%
	Other: UTI	14	7%
	Other: BPH	1	0.5%
Other outcome	None	183	85%
	Other imaging	11	5%
	Other test	2	1%
	Referral	18	8%
	Surgery	2	1%
Subsequent outcomes			
No. of visits for abdominal pain	1	148	69%
	2	53	25%
	3	12	6%
	4	2	1%
	7	1	0.5%
Abdominal pain care conclusion	Not concluded	151	70%
	Concluded	65	30%
Abdominal pain care conclusion category if concluded	Other imaging	6	9%
	Initial medical management	28	43%
	Other medical management	4	6%
	Referral/surgery	27	42%

Relationships among demographic and medical covariates

Several significant relationships were found among covariates of interest. Gender (p=0.033) and initial medical management (p<0.001) varied across the three presenting problems. Namely, men (27/73, 37%) were more likely than women (30/143, 21%) to present with epigastric pain (p<0.05). Regarding medications, patients with epigastric pain were more likely to be prescribed medications of any kind (54/57, 95%) than were unspecified pain patients (72/90, 80%) or RUQ pain patients (50/69, 72%; both p<0.05). They were also the most likely to be prescribed GI medications only (54/57, 95%), followed by RUQ pain patients (32/69, 46%), then by unspecified abdominal pain patients (18/90, 20%; all p<0.05). A combination of analgesic and GI medications was prescribed for unspecified pain (14/90, 16%) and RUQ pain (8/69, 12%) but not epigastric pain (both p<0.05). Prescribing analgesic medications only was most common for unspecified abdominal pain (25/90, 28%), followed by RUQ pain (10/69, 14%), and did not occur at all for epigastric pain (both p<0.05). Other medications (e.g., antibiotics for urinary tract infection [UTI]) were prescribed only for unspecified abdominal pain (15/90, 17%) and not for the other two presenting problems (both p<0.05).

Age (p=0.122), gender (p=0.023), and insurance status (p=0.024) were also related to initial medical management. Specifically, patients prescribed a combination of analgesic and GI medications were younger (M=36.8 years, SD=11.6) than patients prescribed no medications (M=47.2, SD=14.8, p=0.012). Men were more likely than women to be prescribed analgesics (17/73, 23% vs. 18/143, 13%; p<0.05) or GI medications (42/73, 58% vs. 62/143, 43%; p<0.05), while women were more likely to be prescribed no medications (34/143, 24% vs. 6/73, 8%; p<0.05). Uninsured people were more likely than insured people to be prescribed a combination of analgesic and GI medications (15/92, 16% vs. 7/124, 6%, p<0.05). Finally, age was related to insurance status, such that uninsured patients were younger (M=40.1, SD=13.5) than insured patients (M=45.6, SD=16.6, p=0.010). There were no significant relationships between age and gender, age and presenting problem, gender and insurance status, or insurance status and presenting problem.

Demographic and medical covariates in relation to abdominal ultrasound completion and visit resolution

We next examined the relationship of covariates of interest with abdominal ultrasound completion, the primary independent variable. Abdominal ultrasound completion was significantly associated with age (p=0.010), presenting problem (p<0.001), and initial medical management (p=0.032), but not gender (p=0.984) or insurance status (p=0.727). Patients who completed the ultrasound ordered were older (M=47.7 years, SD=15.9) than patients who did not (M=41.6, SD=15.1, p=0.010). Abdominal ultrasounds were more commonly ordered for patients with RUQ pain (40/69, 58%) than patients with epigastric (9/57, 16%) or unspecified abdominal pain (10/90, 11%; both p<0.05). Patients initially prescribed analgesics, GI medications, both analgesic and GI medications, or no medications were similarly likely to undergo abdominal ultrasound (20-36%, all p>0.05). However, patients initially prescribed other medications (namely, for UTI) were less likely to undergo ultrasound; in fact, none of them did so (0%; p<0.05 vs. other groups).

We next examined the relationship of covariates of interest with resolution of visits for abdominal pain in the primary care office, the primary outcome. Conclusion of visits for abdominal pain in the primary care setting was related to the presenting problem (p<0.001) such that it was more common among patients with RUQ pain (33/69, 48%) than for patients with epigastric (14/57, 23%) or unspecified pain (19/90, 21%; both p<0.05). Patients whose abdominal pain visits concluded were slightly older than those whose pain did not resolve (M=46.1, SD=17.1 vs. M=41.1, SD=14.8), but this difference did not reach the threshold of statistical significance (p=0.085). The conclusion of visits for abdominal pain in the primary care setting was not related to gender (p=0.992), insurance status (p=0.269), or initial medical management (p=0.423).

In summary, age, presenting problem, and initial medical management were associated with either abdominal ultrasound completion or pain care completion and therefore represented potential confounds to account for when examining the relationship between the abdominal ultrasound completion and completion of care for abdominal pain.

Abdominal ultrasound completion in relation to care conclusion

**Figure 1:**
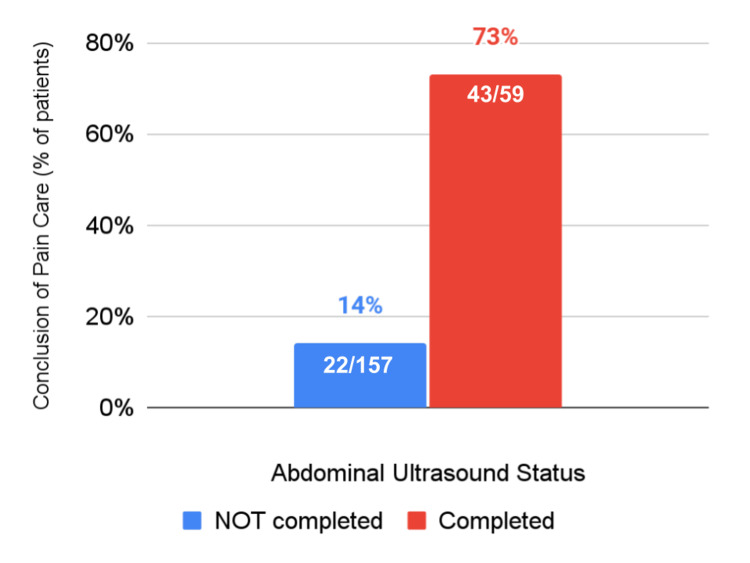
Conclusion of care for abdominal pain in relation to abdominal ultrasound completion

We examined the association of abdominal ultrasound with the pain management outcomes of interest, both alone and when adjusting for covariates. As shown in Table 2, undergoing an abdominal ultrasound was associated with markedly higher odds of conclusion of care for abdominal pain in the primary care office (OR=16.49, 95% CI=7.95-34.21, p<0.001), and this relationship remained significant when adjusting for demographic and medical covariates (OR=17.30, 95% CI=7.04-42.49, p<0.001). Almost three-quarters of people who underwent abdominal ultrasound (43/59, 73%) had conclusion of primary care for abdominal pain versus only 14% (22/157) of patients who did not have abdominal ultrasonography (Figure 1).

**Table 2:** Logistic regression of conclusion of care for abdominal pain in relation to abdominal ultrasound completion Abn, abdominal; US, ultrasound; RUQ, right upper quadrant; GI, gastrointestinal

		B	S.E.	Wald	df	p-Value	OR	95% CI for OR	
								Lower	Upper
Block 1	Abn US completed	2.80	0.37	56.67	1	<0.001	16.49	7.95	34.21
	Constant	-1.81	0.23	62.27	1	<0.001	0.16		
Block 2	Abn US completed	2.85	0.46	38.64	1	<0.001	17.30	7.04	42.49
	Age	0.00	0.01	0.03	1	0.863	1.00	0.98	1.03
	Women vs. men	-0.07	0.41	0.03	1	0.859	0.93	0.42	2.06
	Insured vs. uninsured	0.31	0.40	0.62	1	0.431	1.36	0.63	2.96
	Presenting Abn pain problem			0.05	2	0.977			
	RUQ vs. unspecified	0.11	0.51	0.05	1	0.831	1.12	0.41	3.06
	Epigastric vs. unspecified	0.05	0.61	0.01	1	0.932	1.05	0.32	3.46
	Medical management			2.31	4	0.679			
	Analgesic vs. none	0.90	0.68	1.74	1	0.187	2.46	0.65	9.35
	GI vs. none	0.54	0.59	0.85	1	0.358	1.72	0.54	5.49
	Analgesic and GI vs. none	0.24	0.80	0.09	1	0.765	1.27	0.26	6.10
	Other vs. none	0.96	0.86	1.25	1	0.263	2.61	0.49	14.04
	Constant	-2.65	0.82	10.36	1	0.001	0.07		

Abdominal ultrasound completion in relation to number of visits

**Figure 2:**
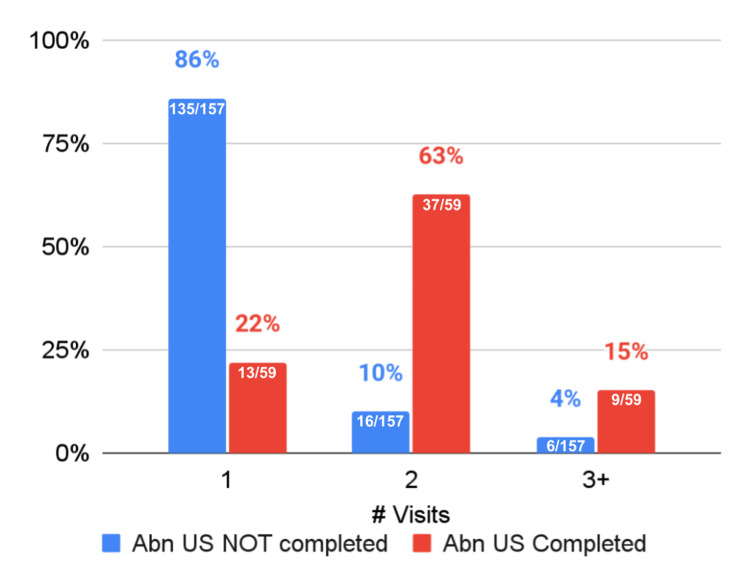
Number of visits for abdominal pain in relation to abdominal ultrasound completion Abn, abdominal; US, ultrasound

As shown in Table 3, patients who underwent abdominal ultrasonography were more likely to have multiple visits for abdominal pain (OR=21.71, 95% CI=10.12-45.57, p<0.001), and this relationship remained when adjusting for covariates (OR=22.66, 95% CI=8.92-57.55, p<0.001). Most people who underwent abdominal ultrasonography (46/59, 79%) had two or more visits for abdominal pain, versus only 14% (22/157) of patients who did not undergo abdominal ultrasonography (Figure 2). Notably, when patients had multiple visits, they typically only had two visits; only 15% (9/59) of patients who completed abdominal ultrasonography had three or more visits, compared with 4% (5/157) of patients who did not complete abdominal ultrasonography.

**Table 3:** Logistic regression of abdominal ultrasound completion in relation to number of visits (2+ vs. 1) Abn, abdominal; US, ultrasound; RUQ, right upper quadrant; GI, gastrointestinal

		B	S.E.	Wald	df	p-Value	OR	95% CI for OR	
								Lower	Upper
Block 1	Abn US completed	3.08	0.39	62.52	1	< .001	21.71	10.13	46.57
	Constant	-1.81	0.23	62.27	1	< .001	0.16		
Block 2	Abn US completed	3.12	0.48	43.07	1	< .001	22.66	8.92	57.55
	Age	0.00	0.01	0.00	1	0.974	1.00	0.98	1.03
	Women vs. men	-0.05	0.42	0.01	1	0.907	0.95	0.42	2.15
	Insured vs. uninsured	0.35	0.41	0.75	1	0.388	1.42	0.64	3.15
	Presenting Abn pain problem			0.10	2	0.949			
	RUQ vs. unspecified	0.02	0.53	0.00	1	0.974	1.02	0.36	2.86
	Epigastric vs. unspecified	-0.17	0.61	0.07	1	0.786	0.85	0.25	2.82
	Medical management			1.84	4	0.766			
	Analgesic vs. none	0.69	0.69	1.01	1	0.315	2.00	0.52	7.68
	GI vs. none	0.52	0.60	0.75	1	0.386	1.68	0.52	5.47
	Analgesic and GI vs. none	-0.01	0.82	0.00	1	0.995	1.00	0.20	4.93
	Other vs. none	0.79	0.85	0.87	1	0.352	2.21	0.42	11.79
	Constant	-2.44	0.83	8.73	1	0.003	0.09		

## Discussion

As this study has shown, there is an association between Hispanic/Latino patients having an abdominal ultrasound and thereafter experiencing conclusion of primary care visits for abdominal pain. Moreover, this association was not driven by potential demographic and clinical confounds, such as age or diagnostic code. A study by Yi et al. showed the utilization of diagnostic imaging in primary care and supported this study’s finding that primary care imaging was not impacted by demographics [11]. There are no comparative studies evaluating how abdominal pain impacts resolution of care of patients in primary care. There are studies supporting that surgeon-performed ultrasonography in the emergency rooms aided in patient satisfaction, but that could be related to prompt evaluation and or diagnosis with imaging or negative imaging results [12].

In this study, patients with abdominal ultrasounds for abdominal pain typically had two visits for abdominal pain, whereas patients without abdominal ultrasounds typically had just one visit. Thus, while ultrasonography was associated with an increase in primary care utilization, that increase was small. The reason that patients with abdominal ultrasounds for abdominal pain had more visits is likely secondary to patients wanting to receive results of their testing. Studies show that diagnostics often reduce uncertainty and thus reduce the overall healthcare cost to the patient and burden to the system overall [13]. This study did not define if a patient was following up for results or because their pain was persistent. This might be a worthwhile future study to investigate when patients were only following up for results of testing versus still having complaints of pain.

Clinical indication is obviously the major indicator when abdominal ultrasonography is ordered. But as seen in other illnesses such as chronic low back pain, patients’ desire for imaging can influence when imaging is ordered [14]. With this notion in mind, another reason that patients may have had more visits when ultrasonography was used is that the patient urged the provider to order additional testing. This aspect was not investigated during the study, and if it had been, a patient's desire for imaging may not be mentioned in the progress notes. Providers may not feel comfortable relaying some of the details of the visit due to the patient’s perception if the progress notes are later obtained by the patient [15].

This study confirms what other studies have noted, that women seek care more often for abdominal pain than men do [16,17]. There is no pointedly clear reason for this disparity noted in research nor does this study explain the causality. Alternatively, the diagnosis of epigastric pain in this study showed that men presented more often with this complaint. To our knowledge, there are no studies that support or oppose this finding. This study noted that men were more likely to receive prescriptions for analgesics for their abdominal pain complaints. There are studies that support similar gender disparity regarding analgesics, but was only noted in the emergency setting and with administration of opioid analgesics [18]. Chen et al. noted that they did not see disparity with administration of non-opioid analgesia in patients who presented to the emergency room for abdominal pain [18].

This study noted that patients who were younger were more likely to receive multiple types of medications (GI and analgesic). Studies show that millennials want medical care that is efficient [19]. This could indicate that they expect a prompter resolution of care and thus suggest that more medications are equivalent to better and more effective care. Gray and Wood noted that prescription medications in the younger population often aid their emotional well-being [20]. The higher amounts of prescription drugs among the younger patients may be related to another study that mentions that younger adults are more likely to wait longer to obtain care due to prior negative healthcare experiences [21]. Thus, this may require more medications to treat the presenting problem.

In this study, uninsured patients were more likely to receive a combination of treatments including both GI-based medical treatment and analgesics. This is presumptively because the providers wanted to treat as many etiologies as possible due to the financial burden of repeated visits for the same medical problem. Many studies demonstrate the inequities of care for the uninsured including clinical outcomes [22,23]. For the purpose of this study, the outcome regarding resolution of pain care after abdominal ultrasonography was equivalent in the insured versus uninsured, as was the number of primary care visits for abdominal pain. This study did not evaluate why these patients had the same outcomes regarding conclusion of care or number of visits. However, it is notable that uninsured people, who commonly face disparities in access to care, had comparable care outcomes in this study in comparison with insured people [24], which may relate to this clinic’s familiarity with uninsured patients or steps the clinic has taken to reduce care barriers.

Limitations

Conclusion of care for abdominal pain was often unknown if the patient did not have additional visits. It was coded as unresolved but could have been resolved and not been documented. Because patients who underwent abdominal ultrasonography tended to have multiple visits, this could bias results in favor of finding an association between abdominal ultrasonography and conclusion of care. The number of patients who had abdominal ultrasound ordered but did not complete it was too small to analyze as their own group; thus, they were grouped with others who did not complete it. The ideal approach would be to examine their outcomes separately and determine the factors that drive ultrasound non-completion.

## Conclusions

In summary, the results of this study of predominantly Hispanic/Latino adults with new complaints of abdominal pain showed that abdominal ultrasonography did impact the resolution of care for abdominal pain in the primary care setting of rural Rio Grande Valley of South Texas, with only a slight increase in primary care utilization versus the hypothesized decrease in need for care. Further studies are indicated to demonstrate whether the benefits of ultrasonography and pain care conclusion outweigh the increased cost associated with ultrasonography and an additional visit. This study also demonstrated that further studies should be performed on the Hispanic/Latino uninsured population to guide physicians in cost-effective and complete care.
